# Supercritical CO_2_-Stable Cementing Materials Based on Vinyl Ester Resin for Maintaining Wellbore Integrity

**DOI:** 10.3390/ma18020244

**Published:** 2025-01-08

**Authors:** Zhong Li, Zhiming Yin, Dingzhao Zhou, Zhiqiang Wu, Daohang Wang, Shuwen Guan, Guangyan Du

**Affiliations:** 1 CNOOC Research Institute Co., Ltd., Beijing 100028, China; lizhong@cnooc.com.cn (Z.L.); yinzhm@cnooc.com.cn (Z.Y.); zhoudzh2@cnooc.com.cn (D.Z.); wuzhq2@cnooc.com.cn (Z.W.); 2 College of Materials Science and Engineering, Zhejiang University of Technology, Hangzhou 310014, China; 2112125158@zjut.edu.cn; 3 SLB Technology Services (CD) Co., Ltd., Chengdu 610200, China; sguan7@slb.com

**Keywords:** carbon sequestration, carbon corrosion, wellbore integrity, cement, vinyl ester resin

## Abstract

Ensuring long-term wellbore integrity is critical for carbon dioxide geological storage. Ordinary Portland cement (PC) is usually used for wellbore primary cementing and plug operation, and set cement is easily corroded by acidic fluids, such as carbon dioxide, in underground high-temperature and high-pressure (HTHP) environments, resulting in a decrease in the mechanical properties and an increase in permeability. In order to achieve long-term wellbore integrity in a CO_2_-rich environment This study introduces materials such as thermosetting vinyl ester resin (TSR), filler composite resin (FCR), and low-cost resin cement (RC). Corrosion experiments were conducted using four materials in 28 days under supercritical carbon dioxide gas and water phase conditions of 60 °C and 10 MPa. The samples were characterized through mechanical property testing machines, core permeability measuring instruments, FTIR, XRD, and SEM. The results proved that after corrosion, PC mechanical properties decreased, the permeability increased, and the microscopic composition and morphology changed greatly. Penetrating corrosion occurs in the sample in the gas phase environment, and propulsive corrosion from outside to inside occurs in the water phase environment. However, TSR, FCR, and RC materials all maintain excellent resistance to carbon dioxide corrosion in gas and water environments. They have higher compressive strength and extremely low permeability compared to ordinary Portland cement. These three materials’ compressive strengths can be maintained around 131, 99, and 58 MPa, and permeability can be stabilized at <6 × 10^−7^, <6 × 10^−7^, and 0.16 mD levels. In summary, the above three materials all show better performance than ordinary Portland cement and are promising alternative materials that can be used in primary cementing and plug operations of carbon dioxide geological storage wells.

## 1. Introduction

**Figure 1:**
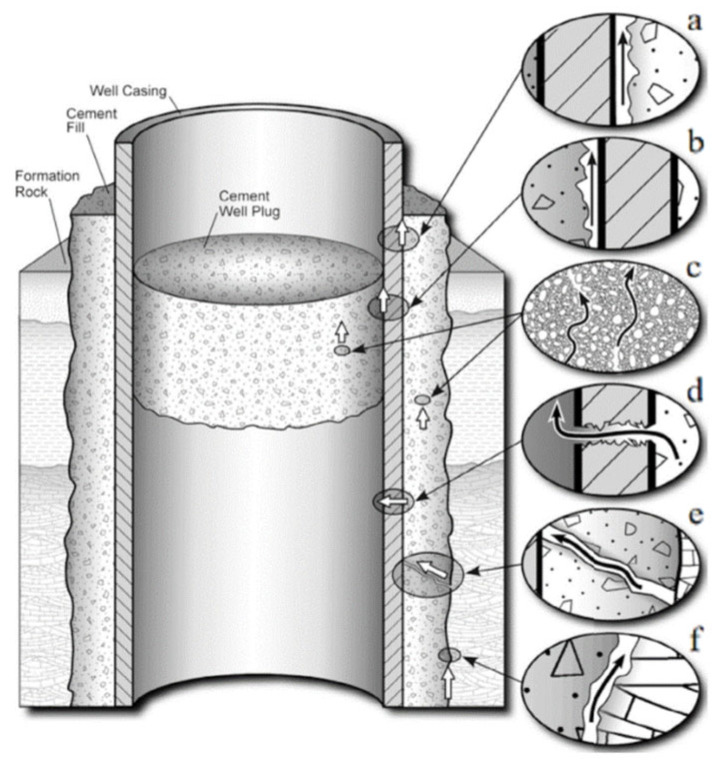
Schematic diagram of possible leakage paths in abandoned wells: (**a**) between casing and cement stone; (**b**) between cement plug and casing; (**c**) through cement stone pore space due to cement stone degradation; (**d**) through casing due to corrosion pipe; (**e**) through cracks in cement stone; (**f**) cracks between cement stone and rock [18].

CCUS (Carbon Capture, Utilization, and Storage) refers to the technology that separates CO_2_ from industrial processes, energy utilization, or the atmosphere and directly utilizes it or injects it into the formation to achieve a permanent reduction in CO_2_ emissions [1]. The main storage sites for CCUS are deep-sea saline aquifers, unminable coal seams, and depleted oil and gas reservoirs. The final step in the process is the permanent storage of CO_2_ [2,3]. Safe and long-term stable storage is a key factor for the success of CO_2_ storage. The annual CO_2_ leakage rate must be kept below 0.1% [4]. Otherwise, CO_2_ leakage will cause damage to groundwater resources, animal and plant life, human health, and other resources. Therefore, ensuring wellbore integrity is critical to successfully storing CO_2_, thereby preventing accidental leakage of CO_2_ to the surface or adjacent permeable layers. It is an indispensable part of ensuring the safe storage of CO_2_ [5]. When CO_2_ is injected and stored, the casing usually needs to be used to prevent fluid leakage and escape, and cement must maintain low permeability during prolonged exposure to reservoir conditions [6,7]. Portland cement is an alkaline porous material, so ordinary Portland cement has problems such as degradation, insufficient chemical resistance and acid resistance, reduced strength, and increased permeability in a CO_2_-rich environment [8,9,10]. In particular, at the HTHP of geological storage, CO_2_ is transformed into the more aggressive supercritical carbon dioxide (scCO_2_) [11]. CO_2_ reacts with formation water to form carbonic acid (H_2_CO_3_) [12]. When H_2_CO_3_ comes into contact with cement stone, a series of chemical reactions will occur, causing corrosion (also known as carbonation) and resulting in changes to the microstructure and macroscopic properties of the cement stone [13], ultimately reducing the compressive strength of the cement stone and increasing porosity and permeability [10,14,15,16,17]. After the cement stone is corroded, the wellbore integrity is destroyed. At this time, the possible migration paths of the fluid are shown in Figure 1.

In recent years, a lot of research has been performed on carbonation-resistant cement materials under CO_2_ storage conditions. For example, basalt powder is used as a supplementary cementitious material in the blend of cementing the CO_2_ storage wellbore to fill the porous cement network, reduce the porosity and permeability of the cement stone, and improve its CO_2_ corrosion resistance [19]. Composite materials from agricultural waste (palm oil fuel ash and rice husk ash) and nanomaterials (nano-silica) are also mixed into a blend to improve CO_2_ corrosion resistance [20,21]. However, such fillers can make the cement difficult to mix, and researchers recommend reducing the water–cement ratio, which can increase the risk of cracking in the cement stone [22,23]. There are also some polymeric additives being added in the mixing fluid of slurry to resist CO_2_ erosion, such as epoxy resin [24,25] and latex [26]. However, the slurry mixing with a polymer has high rheology in surface temperature, which makes it hard to pump in deep oil and gas wells, and poor mixing will lead to dehydration of the stone slurry [27]. It has also been suggested that epoxy resin should be applied to the cement surface as an isolation layer, but the durability of epoxy cement composites will decrease under HTHP conditions of formation [21]. The addition of latex polymers has not provided much improvement over conventional cement [28].

In the harsh environment of carbon sequestration, ordinary Portland cement used to maintain long-term wellbore integrity may not be the best solution due to the various drawbacks of cement stone [29,30]. When it comes to permanently isolating oil and gas wells, more reliable alternative materials are needed to avoid CO_2_ leaks [31]. Therefore, a vinyl ester resin material (TSR) was developed in this study. Compared with ordinary Portland cement, this resin material has the following advantages: low apparent viscosity for easy pumping, adjustable density and viscosity, controllable curing time with “right-angle thickening” curing behavior, wider curing temperature range (0–150 °C), stronger mechanical properties and chemical resistance, better fluid sealing performance, no need for maintenance, and longer service life. These special properties of the TSR resin enable it to withstand the tough downhole environment, ensuring reliable long-term wellbore integrity when facing CO_2_ storage [32]. The composite resin (FCR) was formulated using TSR resin as the matrix and ultrafine barite as the filler. TSR resin was combined with cement slurry to create a cost-effective resin cement (RC) composite. These two types of composite materials represent excellent options for various construction applications.

To simulate carbon sequestration downhole conditions, mechanical property test specimens and standard core samples prepared from four materials, TSR, FCR, RC, and PC, were exposed to the gas and water phase environment of supercritical carbon dioxide at 60 °C and 10 MPa for 28 days. By measuring the permeability, corrosion depth, compressive strength, changes in microcomposition, and microstructure of these four materials before and after corrosion, their comprehensive performance after exposure to CO_2_ for 28 days was evaluated. The aim of this research is to develop several new materials with better performance than ordinary Portland cement and better chemical resistance and to provide a reference for the selection of cementitious materials in primary cementing and remedial jobs in carbon dioxide storage wells.

## 2. Experiments and Methods

### 2.1. Materials

Class G cement was purchased from Shandong Weifang Jiuqi Building Materials Co., Ltd. (Weifang, China). The chemical composition of Grade G well cement is shown in Table S1 in the Supplementary Materials. Vinyl ester resin (DM411-350) was purchased from Changzhou Lebang Composite Materials Co., Ltd. (Changzhou, China). The resin can be produced by ring-opening polymerization of epoxy resin E51 and methyl acrylic acid at 100–120 °C (η = 350 ± 85 m Pa. s). A Styrene–Ethylene–Butylene–Styrene (SEBS) triblock copolymer was purchased from Shanghai Changshi Plasticizing Co., Ltd. (Shanghai, China). Styrene, 3-aminopropyltriethoxysilane, calcium carbonate powder, cobalt naphthenate, and benzoyl peroxide, all of which were analytical grade, were provided by Shanghai Aladdin Reagent Co., Ltd. (Shanghai, China). The inorganic filler is ultrafine barite with a particle size between 3 and 5 μm, and the suspension stabilizer fumed silica had a particle size of about 7 nm. Ultrafine barite and fumed silica were provided by Shanghai Liangjiang Titanium White Chemical Products Co., Ltd. (Shanghai, China). All materials were used as received without purification.

### 2.2. Preparation of Cementitious Materials Agent TSR, FCR, and RC

(1) Thermosetting vinyl ester resin (TSR)

Vinyl ester resins offer superior durability, temperature resistance, and corrosion resistance. They are, therefore, frequently utilized when excellent performance against chemicals and solvents is required. At room temperature, vinyl ester resin and styrene were added with a mass ratio of 1:1 in a round-bottomed flask as the main cementitious materials agent, and then 0.1 wt% of 3-aminopropyltriethoxy was added as the main cementitious materials agent. The silane coupling agent is a common organosilicon compound with a group that can be simultaneously combined with both inorganic (e.g., glass fiber, silica, calcium hydroxide, barite, etc.) and organic materials. After the organic phase was stirred and mixed evenly, 0–10 wt% SEBS plastic particles as the main cementitious materials agent were added, as well as the anti-shrinkage agent and toughening agent of the cementitious material agent. The thermoplastic properties of SEBS enable it to maintain a certain fluidity during the curing process of the resin. When the resin undergoes a curing reaction, the volume will change significantly due to the cross-linking and contraction of the molecular chains. The addition of SEBS can form a phase state different from that of the resin matrix, which can act as a “buffering” mechanism during the curing process and effectively reduce the shrinkage stress. Stir at room temperature for about 5 h until the plastic particles are dissolved and obtain a synthetic resin cementing agent. Curing agents, such as benzoyl peroxide, can be compounded with conventional organic solvents to form a liquid initiator within the initiation temperature according to the requirements of the reaction time and can be reasonably added to the required content.

(2) Filler composite resin (FCR)

The composite resin formulation, designated as FCR, was developed by incorporating a 1% mass fraction of fumed silica and an 80% mass fraction of ultrafine barite into the resin formulation TSR. Barite is a commonly used filler for resin matrix composites with a low price and wide source, and it has excellent chemical stability, is almost insoluble in water and hydrochloric acid, and can stably exist in high temperatures, high pressures, and high concentrations of acid and alkali environments.

(3) Resin cement (RC)

After the prepared slurry was placed into the mixing cup, 50 wt% TSR resin was added directly while maintaining a rotation speed of 6000 r/min. The two components are then stirred for approximately 60 s to ensure thorough mixing.

The production criteria for cement slurry, core samples, and mechanical properties test samples can be found in the Supplementary Materials, showing the B-Sample preparation method and the size.

### 2.3. Carbonation Test

**Figure 2:**
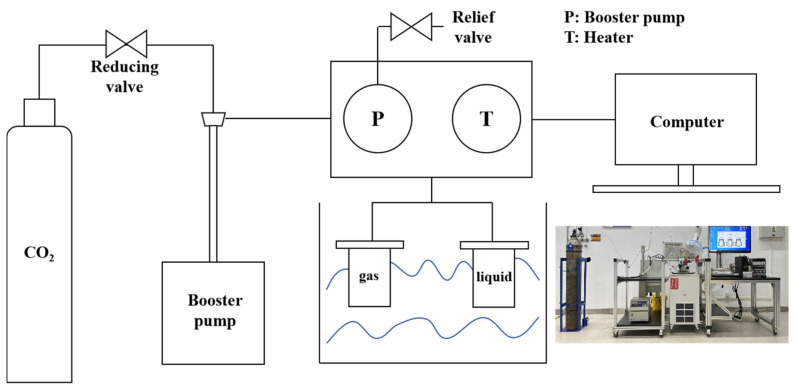
Schematic diagram of static corrosion device of the high-temperature and high-pressure (HTHP) reactor.

Carbonization test standards were referred to as GB/T 50082-2024 [33]. The prepared standard core samples and standard mechanical property test samples of the four materials were placed in an HTHP reactor. A schematic diagram of the apparatus is shown in Figure 2. The reactor is divided into gas phase and water phase environments. The complete set of HTHP reactor equipment is provided by China National Offshore Oil Corporation (Beijing, China). Ultrapure CO_2_ is continuously fed into the pipeline of the apparatus to eliminate air; then, the entire apparatus is sealed, and a pressure pump and water bath are used to set the pressure and temperature of the reactor to 10 MPa and 60 °C, respectively. The reactor was enclosed and placed in water to see if it leaked gas. After confirming that the reactor has no leakage, the temperature and pressure are heated and pressurized, and the temperature and pressure are controlled numerically and kept constant to ensure the repeatability of the experimental results. At this temperature and pressure, the CO_2_ is ultrapure, a critical state. Deionized water is used in the water environment instead of brine because the solubility of CO_2_ in deionized water is much higher than that of ordinary brine [34] to accelerate the dissolution of CO_2_. And, this experiment is a static condition, which is also the case in most carbonation experiments. The reason for this is the distance between the abandoned wells and the injection wells, as saturated CO_2_ brine moves very slowly and negligibly at some distance from the injection wells [35,36]. The reaction was carried out for 28 days under these experimental conditions, and samples were taken at three stages: 7, 14, and 28 days. Attention was paid to the pressure-releasing process, and the pressure-releasing time was kept at about 1 h to avoid sample damage. The carbonized samples were then subjected to mechanical properties, permeability, compositional changes, and micromorphology tests to compare the corrosion resistance of these four materials to supercritical carbon dioxide fluid.

### 2.4. Characterization

Different mass fractions of calcium carbonate powder were added to the basic plugging agent as the weighting agent, and the density of each weighted plugging agent was determined according to the density test method of the specific gravity bottle. The resin viscosity was determined according to GB/T 7193-2008 [37]. The testing instrument used a ZNN-D6 (Qingdao Haitongda Special Instrument Co., Ltd., Qingdao, China) rotational viscometer. Taking the prepared resin or their mixture fluid 350 mL, viscosity was measured using a ZNN-D6 six-speed rotational viscometer. The test conditions and methods are as follows: storage of 4 h at 30 °C in a constant temperature water bath and determination of viscosity under 100 r/min with a ZNN-D6 six-speed rotary viscometer. The density and viscosity values should be repeated 3 times and averaged.

An HTD8040D (Qingdao Haitongda Special Instrument Co., Ltd., Qingdao, China) cementing simulation device is used to monitor the consistency change in the sealing fluid, and the reaction process of cross-linking polymerization between the resin and the curing agent is displayed laterally. It includes control valves, differential pressure threaded sleeves, reaction kettles, consistency meters, and CNC modules. The set temperature and pressure are 75 °C and 15 MPa, respectively.

The thermogravimetric analysis test of the resin-cured body was carried out using the German NETZSCH thermogravimetric analyzer TG 209F3 (NETZSCH, Selb, Germany). The nitrogen purge flow rate is 20 mL·min^−1^, the test temperature range is 25–600 °C, and the heating rate is 10 °C·min^−1^. Three samples were randomly selected and determined three times under the same test conditions.

The compressive strength testing instrument is the Instron5966 high- and low-temperature double-column testing machine, produced by Instron Corporation of the United States. The test standard refers to GB/T 2567-2021 [38]. The compressive strength of four samples in different corrosion time periods is measured. Each data point is measured three times, and the final data are averaged.

The permeability of standard core samples of four materials was measured using the Autolab-2000C HTHP rock physics experimental equipment provided by China National Offshore Oil Corporation (Beijing, China). Pressurize the gas (nitrogen) to create a certain pressure difference at both ends of the core sample, record the inlet and outlet pressure and outlet flow rate of the gas, and finally, calculate the permeability according to Darcy’s law. The permeability of each core sample should be tested three times and averaged.

For the micro-compositional analysis sampling of the four materials, the samples are taken after 28 days of gas and water phase corrosion and are collected with 150-grit sandpaper from the outermost surface according to different corrosion depths of 0–1 mm, 1–2 mm, and 2–3 mm. The sample powder in the corrosion area is analyzed for relevant changes in component analysis. The XRD test uses a Japanese Rigaku Ultima IV X-ray diffractometer (Tokyo, Japan), with a scanning range of 10–70° and a scanning speed of 10°·min^−1^. The FTIR test uses an American Thermo Fisher Scientific infrared spectrometer (Waltham, MA, USA). The test method is KBr tableting, and the scanning range is 4000 to 500 cm^−1^.

Core samples of TSR, FCR, RC, and PC after being corroded in the gas and water phase for 28 days were cut off from the cylindrical surface parallel to the corrosion direction, and relatively flat core blocks were selected for processing and polishing. The processed core blocks were dried overnight in a 60 °C oven, sprayed with gold, and placed into a scanning electron microscope to observe the microscopic morphology (Hitachi S-4700 (II) field emission scanning electron microscope produced by Hitachi, Tokyo, Japan). Then, perform EDS element scanning (Thermo NORAN energy spectrometer produced by American Thermoelectric Company, Chester, PA, USA). The scanning range is 0–1 mm, 1–2 mm, and 2–3 mm random points in the corrosion area. Three random points are selected. The final data are the average of 3 random points. Statistics on EDS element scanning are shown in Tables S2–S5 in the Supplementary Materials.

## 3. Results and Discussion

### 3.1. Density and Viscosity of Plugging Agent TSR

For the leakage point of different wells, considering their formation pressure coefficients are different, it is necessary to adjust their density and viscosity so that the density and viscosity of the plugging agent and the formation pressure coefficient values are close to each other. After entering the micro-seam under the effect of differential pressure, the plugging agent can be precisely controlled by squeezing pressure and squeezing volume so as to realize its residence at the micro-seam.

**Figure 3:**
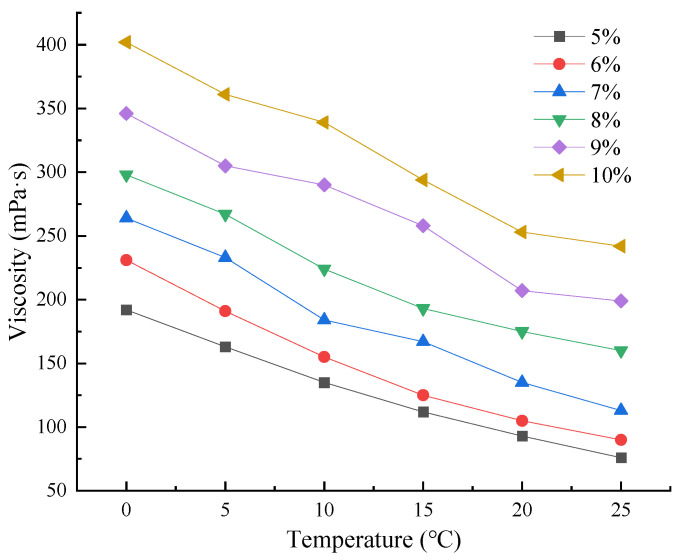
Variation of plugging agent viscosity with temperature for different SEBS contents.

By adding different mass fractions of calcium carbonate powder as an aggravating agent to the plugging agent with a base density of 1.0489 g/mL, a number of groups of plugging agent samples with different densities were designed to study the adjustable range of their densities, which are shown in Table 1. It can be concluded that the experiments successfully adjusted the densities of plugging agents between 1.04 and 1.80 g/cm^3^. The viscosity range of the plugging agent at 0–25 °C was determined by dissolving 5–10 wt% of SEBS plastic in the plugging agent, as shown in Figure 3, to conclude that the viscosity range of the plugging agent at a conventional surface environment is 76–402 mPa·s, which is within the range of pumpable construction. After the plugging agent enters the formation wellbore, the increase in temperature and pressure will result in a lower liquid viscosity compared to the surface.

### 3.2. Vinyl Ester Resin (TSR) Curing Behavior

**Figure 4:**
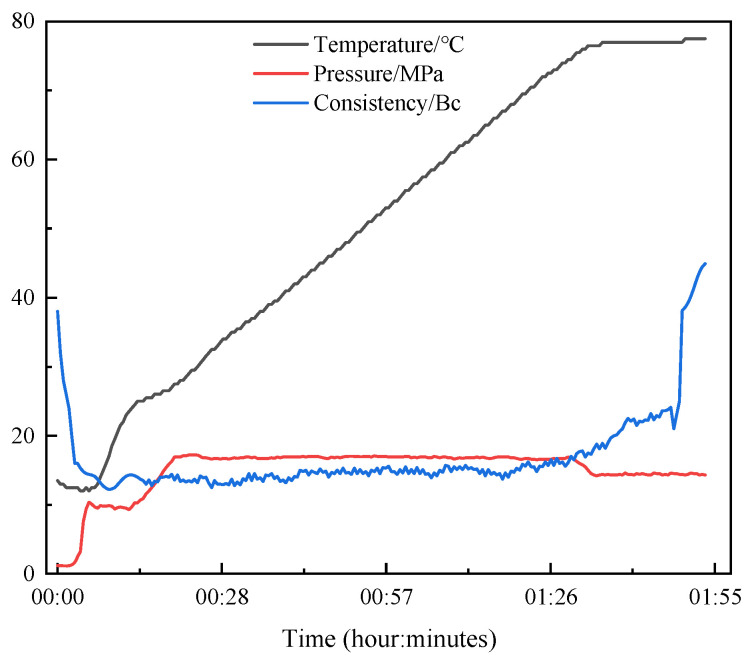
Consistency variation diagram during TSR curing.

The resin curing behavior simulation was conducted under HTHP conditions in the formation. The prepared plugging agent TSR was thoroughly mixed with a BPO solution, and the BPO content was 1 wt% of TSR. Insert the reaction device and record the changes in temperature, pressure, and consistency. As shown in Figure 4, in the initial stage of the reaction, due to the increase in temperature and pressure, the movement speed of liquid molecules increases and the interaction between molecules weakens. This makes it easier for the molecular structure to be destroyed, and the resistance to liquid flow is reduced, thus reducing the viscosity [39]. As the reaction time prolongs, the resin consistency maintains a stable fluctuation state for up to 1.5 h. After 1.5 h, the induction period ends, and the acceleration period begins. Free radical polymerization of unsaturated double bonds occurs in the resin system, resulting in a sudden increase in consistency [40,41]. To avoid damaging the instrument, stop the reaction immediately. The curing behavior of the TSR system exhibits “right-angle thickening” characteristics. This feature ensures smooth pumping and flow of the resin system in the formation and wellbore, thus ensuring the safety of cementing operations.

### 3.3. Thermal Stability of the TSR-Cured Body

**Figure 5:**
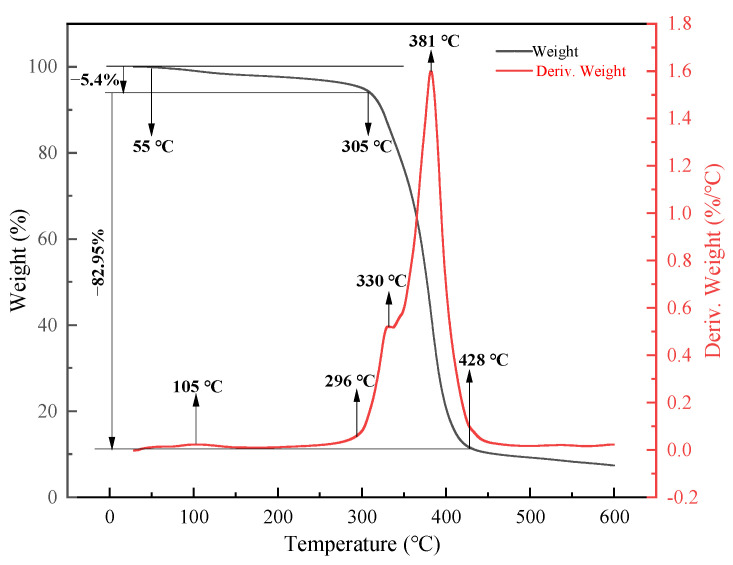
TG and DTG diagram of the TSR-cured body.

Figure 5 shows the TG and DTG curves of a fully cross-linked TSR-cured body in a nitrogen atmosphere. Two weight loss steps (105–305 °C and 305–428 °C) can be observed for the TSR-cured body. It can be seen in the figure that the thermal decomposition temperature of the TSR-cured body is approximately 300 °C, and the weight loss rate of the material before 300 °C does not exceed 6%. The initial decomposition temperature and maximum decomposition temperature of the TSR-cured body are 305 °C and 428 °C, respectively. Degradation of unreacted curing agents or oligomers and polymer chains occurs at 105 °C and 381 °C, respectively. This shows that they have excellent temperature resistance, and the material will not be damaged under normal working conditions.

### 3.4. Changes in Compressive Strength

**Figure 6:**
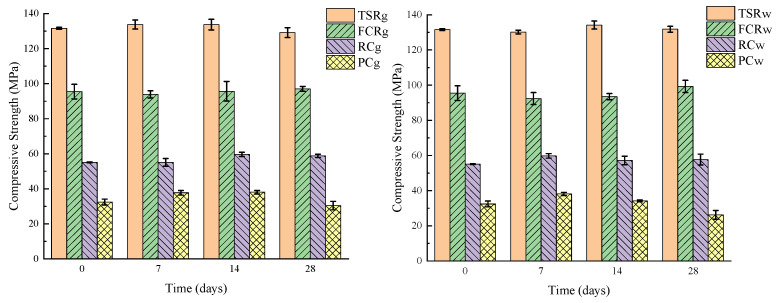
Changes in the compressive strength of materials before and after corrosion in gas phase and water phase environments.

Compare the compressive strength values of samples exposed to gas and water environments with the initial samples, record the data changes, and evaluate the material’s resistance to supercritical carbon dioxide through the changes in compressive strength. The data changes are shown in Figure 6 and Table 2. As can be seen in the graphs, the mechanical properties of TSR and FCR are almost unchanged after corrosion by carbon in the gas and water phase environments, indicating excellent resistance to supercritical carbon dioxide corrosion, and the compressive strengths of the two are much higher than those of the ordinary Portland cement. The compressive strengths of RC and PC first increase and then decrease. The cement stone is carbonized in the early stages of the reaction and CO_2_ reacts with Ca(OH)_2_ and C-S-H to form a high-density corrosion product, CaCO_3_, which fills the pore and crack structure within the cement stone, resulting in an increase in the compressive strength of the cement stone. Later, through leaching and decalcification, the CaCO_3_ produced continues to react with CO_2_ and H_2_O to form soluble Ca(HCO_3_)_2_. Ca(HCO_3_)_2_ is continuously transported out of the cement stone matrix under the action of water, leaving only amorphous silica gel without cementing ability, resulting in a decrease in the mechanical properties of RC and PC [10,42]. Among them, the loss of strength is more severe in the water environment. Although the compressive strength of RC is not as good as the two types of resin materials, its corrosion resistance and mechanical properties are also better than PC, making it a better choice. 

### 3.5. Permeability Changes

The changes in the permeability data of the four materials are shown in Table 3, and the magnitudes of permeability vary widely. The permeability of PC reached 16.70 mD, and the permeability increased after 28 days under both gas and water environment corrosion. The permeability of the sample in the water environment increased more significantly. The permeability of PC and RC decreases in the early stage of corrosion. The principle is the same as the increase in compressive strength, which produces denser CaCO_3_-filled cement microcracks, resulting in a slight decrease in permeability. Finally, permeability increases due to leaching and decalcification.

To achieve effective and safe geological storage of carbon dioxide, the permeability of Portland cement and sealing systems should not exceed 0.1 mD [43]. The permeability of conventional cement appears to be well above this limit, so the effect of carbon corrosion on cement sealing should be fully considered in CCUS projects. The permeability of RC is lower than PC and remains relatively stable. Resin-based materials have extremely low permeability and are very stable and almost unaffected by carbon corrosion, providing a strong guarantee of achieving inter-layer isolation of carbon dioxide.

### 3.6. Sample Microscopic Composition Analysis

#### 3.6.1. Fourier-Transform Infrared Spectroscopy (FTIR)

**Figure 7:**
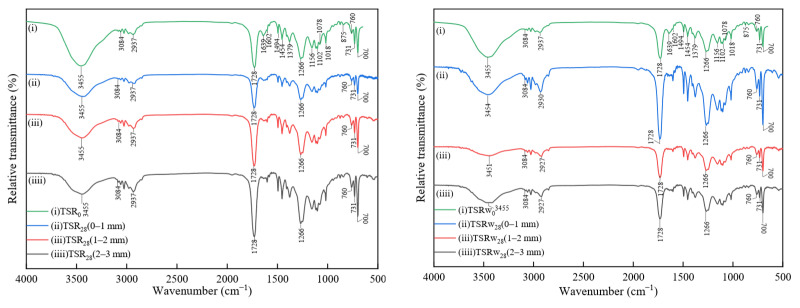
FTIR spectrum at different depths of TSR samples after 28 days of gas phase and water phase corrosion.

**Figure 8:**
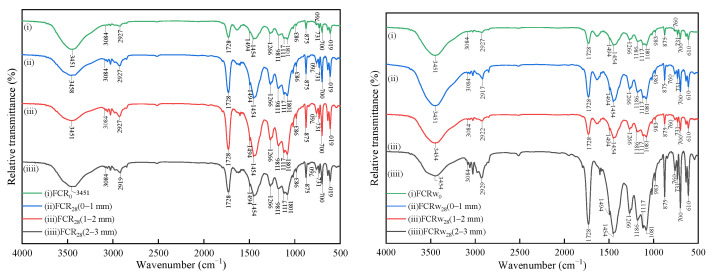
FTIR spectrum at different depths of FCR samples after 28 days of gas phase and water phase corrosion.

Figure 7 and Figure 8 show the FTIR spectra of TSR and FCR samples at different depths after gas phase and water phase corrosion, respectively. For pure resin TSR_0_, the stretching vibration of the hydroxyl group (-OH) is located at 3455 cm^−1^. The stretching vibration of the carbon–hydrogen bond (C-H) of saturated hydrocarbons is located at 2937–3084 cm^−1^, with 3–4 absorption peaks, and the complete vibration of C-H is located at 1454 cm^−1^ and 1379 cm^−1^. Because the vibrational energy levels of the C-H bonds in -CH_2_- and -CH_3_ are not very different, their absorption peaks overlap to form a mixture, and the peak bands are difficult to distinguish. The absorption spectrum of the ester group is the strongest, at 1728 cm^−1^, which is the stretching vibration of the carbonyl group (C=O). The ether oxygen bond (C-O-C) in the ester group has two absorption peaks, namely, the symmetric stretching vibration (ʋ_s_) and the asymmetric stretching vibration. The stretching vibration (ʋ_as_) is located at 1266 cm^−1^, 1156 cm^−1^, and 1102 cm^−1^, respectively. The stretching vibration of the unsaturated double bond (CH=CH) is located at a weak absorption peak at 1639 cm^−1^. The vibration of the benzene ring has two vibrational bands of the conjugated system appearing at 1602 cm^−1^ and 1494 cm^−1^, respectively. The resin is a bisphenol A-type vinyl ester resin with a 1,4-disubstituted benzene ring, so it has medium-strong absorption peaks at 875 cm^−1^ and 731 cm^−1^. 1078 cm^−1^ is the stretching vibration peak of the secondary alcohol, and 1018 cm^−1^ is the stretching vibration peak of the primary alcohol. Values of 700 cm^−1^ and 760 cm^−1^ are the out-of-plane bending vibrations of hydrogen on the monosubstituted benzene ring [44,45]. For the filler composite resin FCR_0_, the infrared vibrational peak of the organic corresponding group of the resin corresponds one to one with that of the pure polyester resin. Among them, the strong absorption peaks in the region of 1081 cm^−1^, 1117 cm^−1^, and 1186 cm^−1^ are the stretching vibrations of oxygen–sulfur bonds. The two strong absorption peaks at 610 cm^−1^ and 983 cm^−1^ are bending vibrations of oxygen–sulfur bonds [46].

After 28 days of carbon corrosion of TSR and FCR in gas and water environments, the infrared spectrum shows no obvious difference from that of the uncorroded initial sample. It shows that these two materials have strong resistance to supercritical carbon dioxide. In the compressive strength and permeability measurements, the two materials also show excellent stability, extremely high mechanical properties, and low gas tightness.

#### 3.6.2. X-Ray Diffraction (XRD)

**Figure 9:**
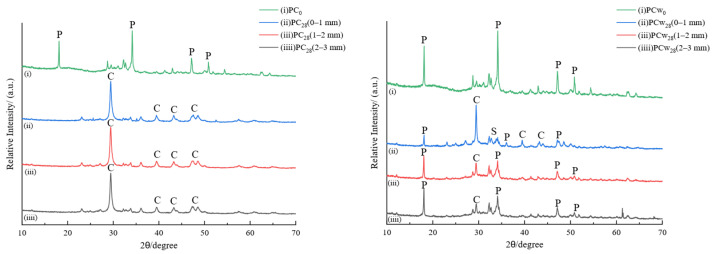
XRD spectrum at different depths of PC samples after 28 days of gas phase and water phase corrosion.

**Figure 10:**
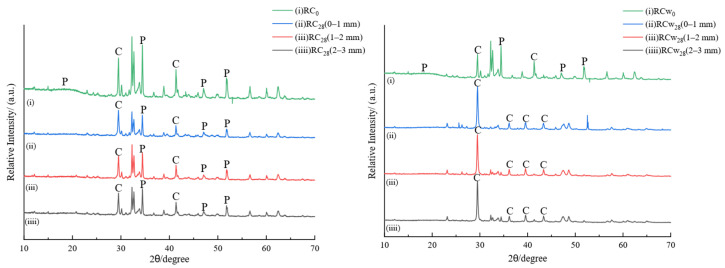
XRD spectrum at different depths of RC samples after 28 days of gas phase and water phase corrosion.

Figure 9 and Figure 10 show the XRD spectra of PC and RC sampled at different depths after gas phase and water phase corrosion, respectively. P represents calcium hydroxide, C represents calcium carbonate, and S represents silica. The main crystalline phase of uncorroded cement PC_0_ is Ca(OH)_2_, and the corresponding crystallographic diffraction angles are 18.00°, 34.19°, 47.10°, and 50.80°. As hydrated calcium silicate changes in many forms with the calcium-to-silicon ratio, there is no obvious diffraction peak. XRD spectra of cement samples have different corrosion depths during gas phase corrosion. The diffraction peaks of Ca(OH)_2_ in the three spectra, (ii), (iii), and (iii), disappear almost completely and are replaced by carbonation of Ca(OH)_2_. The main product after the reaction is calcite-like CaCO_3_. This is in agreement with the previous phenolphthalein color development experiment. Supercritical carbon dioxide penetrates deeply into the cement matrix, and carbonation corrosion occurs simultaneously inside and outside the cement core sample. According to the phenolphthalein color development experiment of water phase corrosion cement samples, it can be seen that relatively uniform corrosion occurs from the outside to the inside of the contact surface. The comparison results show that the characteristic diffraction peak of calcium carbonate appears in the corrosion area of 0–1 mm on the surface of the cement sample, and the intensity of the diffraction peak of Ca(OH)_2_ is significantly lower than that of the uncorroded cement stone. It can be seen that carbonation has occurred on the surface of the cement stone at this time. The characteristic diffraction peak of Ca(OH)_2_ in the 1–2 mm corrosion layer is higher than the surface of the corrosion layer, and the characteristic diffraction peak of calcite is lower than the surface of the corrosion layer. The corrosion layer of 2–3 mm shows the characteristics of uncorroded cement.

For the uncorroded RC_0_, the single strong peak of Ca(OH)_2_ at 18° is weakened. As the polyester resin and cement are evenly mixed, the characteristic diffraction peaks of Ca(OH)_2_ and CaCO_3_ in the cement system are shifted. After the RC sample was corroded by supercritical carbon dioxide, the cross-section of the sample in the gas and water phase environments did not develop any color when exposed to phenolphthalein. This is consistent with the XRD spectrum in Figure 8. Carbonation occurs in the 0–1 mm, 1–2 mm, and 2–3 mm corrosion regions of the gas and water samples, that is, the Ca(OH)_2_ component decreases and the CaCO_3_ component increases, and when the degree of carbonation of the water phase environment is higher, the diffraction peak of Ca(OH)_2_ almost completely disappears. The intensity of the characteristic diffraction peaks corresponding to different corrosion depths is also almost the same, and the overall carbonation degree of the surface is relatively uniform. This also confirms the increase in compressive strength and decrease in permeability of the RC samples mentioned above.

### 3.7. Observation of Sample Micromorphology

**Figure 11:**
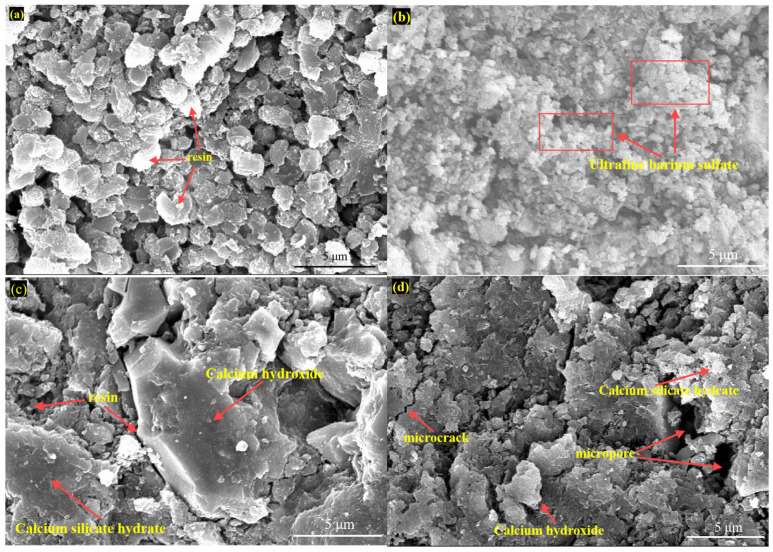
SEM images of four types of core samples without corrosion: (**a**) Thermosetting vinyl ester resin (TSR); (**b**) Filler composite resin (FCR); (**c**) Resin cement (RC); (**d**) Polland Cement (PC).

Figure 11a, Figure 11b, Figure 11c, and Figure 11d show SEM plots of uncorroded samples of TSR, FCR, RC, and PC, respectively. A large number of granular resin polymers can be clearly seen from the SEM images of TSR. These polymers exhibit tight arrangement and uniform particle size, constituting the main structure of the sample. They are intertwined with each other to form a strong and stable skeleton that provides good physical properties for the TSR resin-cured body. Compared with TSR, in addition to a large number of granular resin polymers, many ultrafine barium sulfate fillers with small particle sizes were also observed in FCR. These ultrafine fillers are interspersed and adhered to a large number of resin polymers, making the whole structure more compact and uniform. Ultrafine barium sulfate has a size of 3000 mesh, that is, a particle size of about four microns, and its tiny size allows it to better fill between resin polymers. The SEM image of RC shows the presence of hydrated calcium silicate gel matrix and plate calcium hydroxide crystals, which constitute the main part of the cement matrix. In addition, the granular resin polymers partially filled between the cement matrix can also be observed, which are tightly bound to the cement matrix and together constitute the overall structure of the sample. SEM images of PC uncorroded samples show that the uncorroded cement is mainly composed of hydrated calcium silicate gel matrix, plate calcium hydroxide crystals, and a small number of micropores and microcracks.

**Figure 12:**
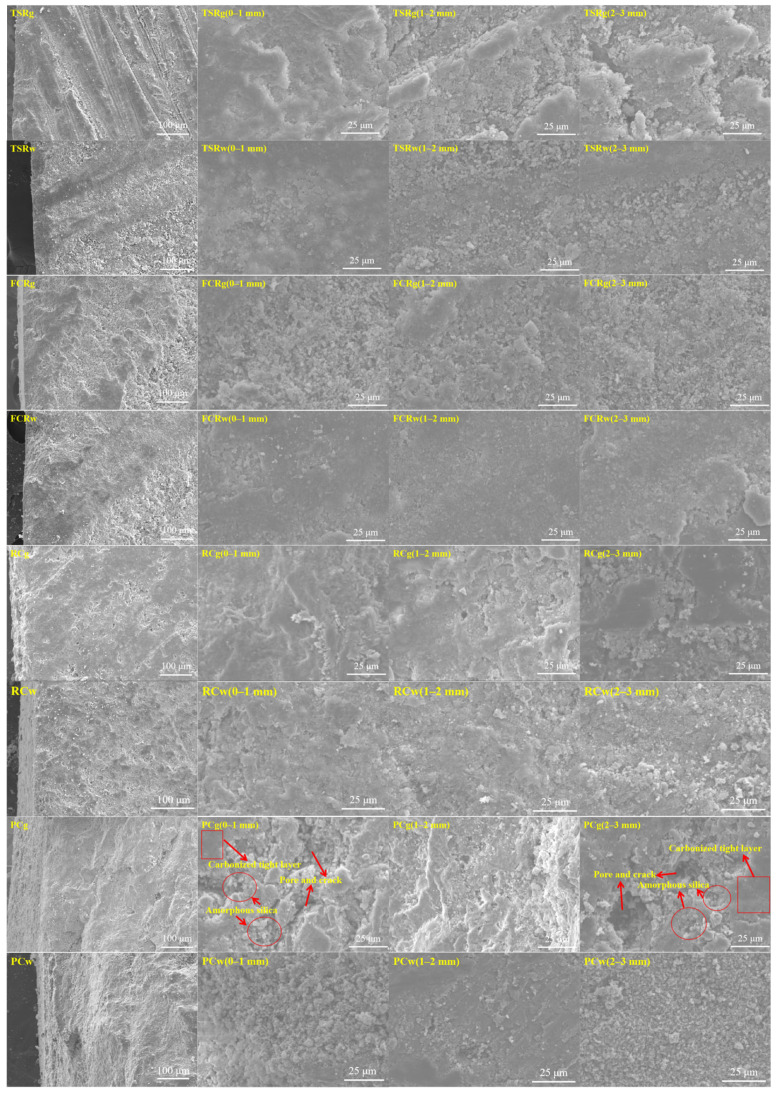
SEM images of cross-sections at different corrosion depths after 28 days of gas phase and water phase corrosion of core samples of four materials.

The SEM images of core samples of four materials at different corrosion depths after 28 days of gas phase and water phase corrosion are shown in Figure 12. After 28 days of corrosion of TSR resin in supercritical carbon dioxide in the gas phase and water phase, SEM images showed no obvious changes in 0–1 mm, 1–2 mm, or 2–3 mm regions, and the structure was dense. This proves its excellent corrosion resistance, consistent with its stable and extremely low gas phase permeability. Under high temperature and pressure, TSR resin core samples showed excellent performance without significant signs of corrosion and maintained a tight structure, further verifying their excellent performance in a supercritical carbon dioxide environment.

After 28 weather and water phase corrosion tests, FCR core samples have excellent corrosion resistance in a supercritical carbon dioxide environment. SEM images showed that the structure of 0–1 mm, 1–2 mm, and 2–3 mm regions was dense, and there was no significant change. The ultrafine barite is evenly filled in the polymer resin matrix, and the gas phase permeability is extremely low, which verifies the excellent performance of the FCR core in extreme environments.

After the corrosion of RC core samples by 28 weather phase and water phase, the SEM image shows that the structure is stable, the gas phase permeability is higher than TSR and FCR but lower than PC, and the sealing property is strong. Calcium hydroxide carbonized into more dense calcium carbonate; the XRD pattern confirmed that the calcium hydroxide peak weakened and that the calcium carbonate peak increased. RC does not change color in the presence of phenolphthalein after carbon corrosion, indicating a decrease in alkalinity.

PC has more pore structures than the other three materials. In PC vapor corrosion, supercritical carbon dioxide has strong fluidity, which leads to integral and irregular penetration corrosion. In the 0–1 mm and 2–3 mm regions, obvious leaching decalcification occurs, and the dense calcium carbonate generated by carbonization is transformed into soluble calcium bicarbonate and is then detached from the PC matrix, leaving only amorphous silica gel with no cementation ability and a large number of pores and cracks, and slight carbonization occurs in the 1–2 mm region. In an aqueous corrosion environment, the structure of PC becomes loose due to slight leaching decalcification at 0–1 mm layer, and the gap between particles increases in SEM images. In the 1–2 mm layer, carbonization is dominant, transforming the calcium hydroxide in this region into a more compact calcite calcium carbonate structure. SEM images show that the morphology is denser and the pores are significantly reduced. As for the 2–3 mm layer, since the dissolved carbon dioxide in the water has not been touched, the cement in this area remains in its original state, and its structure is basically the same as the initial sample.

## 4. Conclusions

In this study, four types of materials, thermosetting vinyl ester resin (TSR), filler composite resin (FCR), resin cement (RC), and Portland cement (PC), were introduced to simulate long-term corrosion resistance experiments under CO_2_ geological storage conditions. The environment of 60 °C and 10 MPa is divided into the supercritical carbon dioxide gas phase and water phase corrosion. The corrosion condition is static corrosion, and the corrosion time is up to 28 days. Experimental evaluations, including compressive strength, permeability, microscopic components analysis, and micromorphology observation, were conducted on the four materials after corrosion. The results show that after long-term carbon corrosion under tough conditions, PC shows a decrease in mechanical properties and an increase in permeability, which is exactly the problem caused by conventional primary cementing. TSR, FCR, and RC show a higher compressive strength and extremely low permeability. The compressive strength of the three materials can be maintained at around 131, 99, and 58 MPa, and the permeability can be stabilized at <6 × 10^−7^, <6 × 10^−7^, and 0.16 mD. FTIR and XRD micro-compositional analysis, SEM morphology observation, EDS elemental scanning, and other data also show their excellent corrosion resistance in supercritical carbon dioxide. All are significantly better than traditional cementitious materials and can provide support for the safe and long-term stable operation of CO_2_ geological storage projects. These findings make these kinds of materials good candidates for lost circulation, zonal isolation, plug and abandonment, casing leak repair, etc. A potential drawback is the large application of TSR resin materials, which could be constrained partly due to their cost. When assessing durability, it is imperative to conduct corrosion resistance tests across a broader spectrum of temperatures and pressures over an extended duration. Furthermore, corrosion media can encompass other typical gaseous corrodents, such as ammonia, hydrogen chloride, hydrogen sulfide, or their combinations.

## Figures and Tables

**Table 1:** Results of density adjustment for different samples.

*w* (talcum carbonate) %	0	20	50	85	126
Density (g·cm^−3^)	1.04	1.20	1.40	1.60	1.80

**Table 2:** Changes in the compressive strength of materials before and after corrosion.

Samples	Initial Strength/MPa	Strength After Corrosion for 7 Days/MPa	Strength After Corrosion for 14 Days/MPa	Strength After Corrosion for 28 Days/MPa
TSRg	131.57	133.69	133.69	129.12
TSRw	131.57	130.2	134.17	131.8
FCRg	95.46	93.87	95.66	97.04
FCRw	95.46	92.37	93.45	99.24
RCg	55.07	55.08	59.67	58.76
RCw	55.07	59.8	57.15	57.68
PCg	32.44	37.72	38.07	30.41
PCw	32.44	38.16	34.2	26.24

**Table 3:** Permeability changes of materials before and after corrosion in gas phase and water phase environments.

Samples	Initial Permeability/mD	Permeability After Corrosion for 7 Days/mD	Permeability After Corrosion for 14 Days/mD	Permeability After Corrosion for 28 Days/mD
TSRg	<6 × 10^−7^	<6 × 10^−7^	<6 × 10^−7^	<6 × 10^−7^
TSRw	<6 × 10^−7^	<6 × 10^−7^	<6 × 10^−7^	<6 × 10^−7^
FCRg	<6 × 10^−7^	<6 × 10^−7^	<6 × 10^−7^	<6 × 10^−7^
FCRw	<6 × 10^−7^	<6 × 10^−7^	<6 × 10^−7^	<6 × 10^−7^
RCg	0.16	0.19	0.09	0.15
RCw	0.16	0.14	0.09	0.16
PCg	16.70	15.51	15.22	17.45
PCw	16.70	13.53	17.89	19.27

## Data Availability

The original contributions presented in this study are included in the article/Supplementary Material. Further inquiries can be directed to the corresponding author.

## Supplementary Materials

The following supporting information can be downloaded at https://www.mdpi.com/article/10.3390/ma18020244/s1, Figure S1: Appearance of four types of core samples after corrosion in gas phase and water phase environments for 28 days; Figure S2: Phenolphthalein test of PC (top) and RC (bottom) specimens uncorroded (left), corroded for 7 days in the vapor (middle) and aqueous (right) phases; Table S1: Chemical composition of grade Class G cement used in this study; Table S2: Elemental content of TSR at different corrosion depths after 28 days of gas phase and water phase corrosion; Table S3: Elemental content of FCR at different corrosion depths after 28 days of gas phase and water phase corrosion; Table S4: Elemental content of RC at different corrosion depths after 28 days of gas phase and water phase corrosion; Table S5: Elemental content of PC at different corrosion depths after 28 days of gas phase and water phase corrosion. Refs. [38,47] are cited in the Supplementary Materials.
