# Supplementary material for: Supercritical CO2-Stable Cementing Materials Based on Vinyl Ester Resin for Maintaining Wellbore Integrity
Source: Materials (Basel). 2025 Jan 8;18(2):244. doi: 10.3390/ma18020244 (PMC11767227; doi:10.3390/ma18020244)
Supplement: Supplementary file 1 [file materials-18-00244-s001.zip › materials-3332015-supplementary.pdf]

Supporting Information

Supercritical CO_2-Stable Cementing Materials Based on Vinyl Ester Resin for Maintaining Wellbore Integrity

Zhong Li [1], Zhiming Yin [1], Dingzhao Zhou [1], Zhiqiang Wu [1], Daohang Wang [2], Shuwen Guan [3] and Guangyan Du [2,*]

[1] CNOOC Research Institute Co., Ltd., Beijing 100028, China; lizhong@cnooc.com.cn (Z.L.); yinzhm@cnooc.com.cn (Z.Y.); zhoudzh2@cnooc.com.cn (D.Z.); wuzhq2@cnooc.com.cn (Z.W.)
[2] College of Materials Science and Engineering, Zhejiang University of Technology, Hangzhou 310014, China; 2112125158@zjut.edu.cn
[3] SLB Technology Services (CD) Co., Ltd., Chengdu 610200, China; sguan7@slb.com
* Correspondence: dugy@zjut.edu.cn

S1. Class G cement composition

Table S1. Chemical composition of Class G cement used in this study.

**Compound**	**Content (wt %)**
Magnesium oxide (MgO)	2.99
Sulfur trioxide (SO_3)	1.94
Loss on ignition	1.59
Insoluble residue	0.57
Tricalcium ailicate	58.65
Tricalcium aluminate	1.12
Tetracalcium aluminate ferrite + 2 times tricalcium Aluminate	16.64
Total alkali as Na_2O	0.60

The composition and proportion of G grade oil well cement used for material preparation in this paper are shown in Table S1.

S2. Sample preparation method and size

S2.1. Portland cement (PC)

The preparation of neat cement refers to the API 10-B (2013) standard [33] and the water–cement ratio is fixed at 0.44. When preparing the slurry, weigh the required mass of the mixing water into a mixing cup, start the high-speed mixer, and keep the speed at 4000 r/min. Within 15 seconds, evenly add cement into the mixing cup and dissolve it in the mixing water, and then continue stirring at a high speed of 12000 r/min for 35 seconds to fully mix the slurry. Pour the prepared slurry into the polytetrafluoroethylene mold and vibrate for 30 seconds to release the air bubbles entrained in the slurry. It is then placed in an oven at a constant temperature of 60 °C and cured for 24 hours. Then, remove it from the mold and place it in salt water containing 0.5 mol/L NaCl to cure for 28 days to fully hydrate the cement sample. The curing temperature was set at 60 °C. After curing, the cement stone samples were repeatedly washed with deionized water and ethanol to remove surface impurities and salts and then dried for later use.

S2.2. Resin cement (RC)

The configuration of the slurry in resin cement is the same as mentioned above. From the point of view of constructability, cost reduction, and efficiency improvement, the optimum synergistic performance can be obtained when the polyester resin addition amount is 50 % of the slurry mass. After the prepared slurry is poured into the mixing cup, a certain amount of polyester resin is

added directly at a speed of 6000 rpm and stirred for about 60 seconds to mix the two completely. The prepared resin cement is poured into the polytetrafluoroethylene mold without vibration to remove air bubbles. It is placed in a constant temperature oven at 60 °C to cure for 24 hours and is then demolded and removed. The subsequent curing conditions and cleaning procedures are the same as for cement.

S2.3. Thermosetting vinyl ester resin (TSR) and filler composite resin (FCR)

The TSR formulation uses a conventional peroxygen initiator, benzoyl peroxide. After mixing, it is poured into the mold and placed in a thermostatic oven at 60 °C to cure for several hours. When fully cured, it is demolded and removed without further maintenance. To further reduce the cost of the TSR, inexpensive ultrafine barite is selectively introduced as a filler, and fumed silica as a stabilizer. After several orthogonal trials, the optimum formulation for overall performance was obtained: add 1 wt% fumed silica and 80 wt% ultrafine barites to the TSR formulation, stir thoroughly, and mix evenly to obtain FCR formulation. The concentration of the initiator type and the sample preparation process are the same as for the TSR.

S2.4. Sample size and reference standard

The sample sizes of the four materials are divided into two types: standard core samples (standard reference GB/T 29172-2012 [1], diameter 25 mm × height 50 mm), which are used for subsequent measurement of core permeability changes, microscopic composition, and morphology observation, and standard

mechanical property test samples (standard reference GB/T 2567-2021 [2], diameter 10 mm × height 20 mm), which are used to measure the change in compressive strength before and after corrosion.

S3. Apparent diagram of sample carbon after corrosion

The appearance of the sample after 28 days of carbonation in the gas and water phase environments is shown in Figure S1.

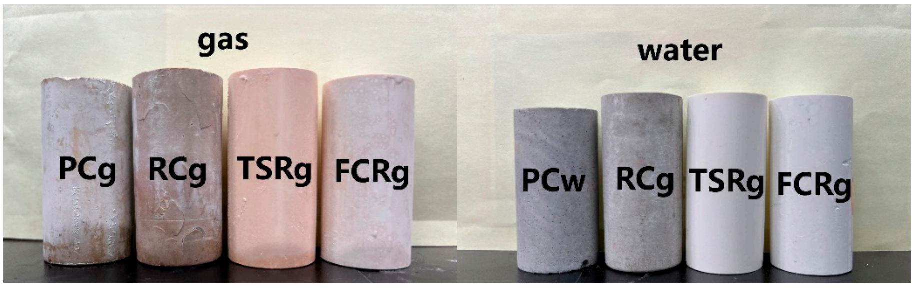

Figure S1. Appearance of four types of core samples after corrosion in the gas phase and water phase environments for 28 days.

S4. Phenolphthalein corrosion depth test

To analyze the depth of corrosion of the sample, the phenolphthalein solution discolors when exposed to alkali if the pH exceeds 8.2. A 1% by mass phenolphthalein alcohol solution is applied evenly to the corroded area. The non-corroded part of the cement stone contains a large amount of alkali. With calcium hydroxide, the cross-section of the sample will appear dark purple on the inside, the corroded area will not discolor due to calcium hydroxide consumption, and there will be a clear zone boundary at the corrosion front.

However, the actual situation is that the PC sample in the gas phase environment is not uniform corrosion from outside to inside. Since carbon dioxide is supercritical, its density is close to that of a liquid, but its fluidity and

diffusivity are equivalent to that of a gas. Therefore, the corrosion of PC samples in the gas phase environment is not uniformly promoted from the outside to the inside, but irregular fluidity corrosion is carried out inside the PC matrix, and the corrosion depth cannot be measured according to this method. In an aqueous environment, the PC sample is completely immersed in water, and the carbon dioxide dissolved in water is uniformly corroded from the outside to the inside. The above situation is illustrated in Figure S2, which shows the phenolphthalein test of the PC and RC samples after no corrosion (left) and after corrosion in the gas phase (middle) and water phase (right) for 7 days.

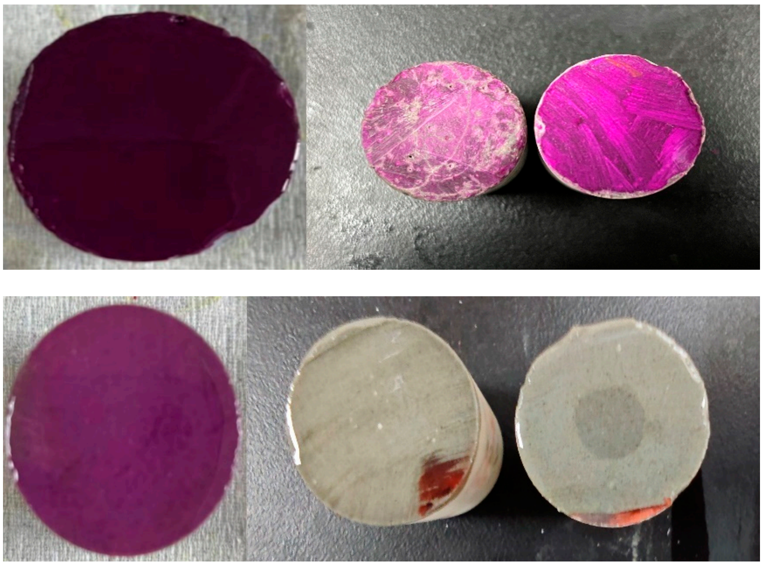

Figure S2. Phenolphthalein test of PC (**top**) and RC (**bottom**) specimens uncorroded (**left**), corroded for 7 days in the vapor (**middle**), and aqueous (**right**) phases.

RC does have a discoloration reaction when it comes into contact with phenolphthalein in the absence of corrosion, but the degree of discoloration is much lighter than that of PC. However, once RC has been subjected to corrosion treatment in a gas or water phase environment, it will no longer show

significant discoloration. This means that it is not possible to calculate the corrosion depth of RC by simply observing the color change. TSR and FCR are two kinds of materials; because they belong to the category of organic polymer materials, they do not have the characteristics of color rendering, so there will be no color reaction in the experiment.

S5. Sample EDS test

The summary results of the EDS elemental scanning analysis of TSR, FCR, RC, and PC are shown in Tables S2–S5. TSR is an organic high molecular polymer, and only two elements, C and O, are selected for scanning statistics. FCR is ultrafine barite, and the main component is $BaSO_4$. Therefore, four elements, C, O, S, and Ba, were selected for scanning statistics. For RC and PC, four elements, C, O, Si, and Ca, were selected for scanning statistics.

The internal elemental composition of TSR and FCR samples showed remarkable stability after corrosion in both gaseous and aqueous environments. By EDS scanning at different depths, it was found that the atomic number proportion of C, O, S, Ba, and other elements did not change significantly compared with the uncorroded samples, which strongly proved the superior properties of these two materials as polymers. They are able to maintain stable chemical properties in harsh corrosive environments and do not react with other components in the surrounding environment, thus demonstrating a strong resistance to supercritical carbon dioxide corrosion.

Table S2. Elemental content of TSR at different corrosion depths after 28 days of gas

phase and water phase corrosion.

Elements	C		O	
**Proportion /%**	**Weight**	**Atom**	**Weight**	**Atom**
TSR_0	67.42	73.38	32.58	26.62
TSRg (0~1mm)	70.25	78.31	22.12	18.51
TSRg (1~2mm)	76.58	82.49	20.00	16.17
TSRg (2~3mm)	76.11	81.94	20.87	16.87
TSRw (0~1mm)	76.06	81.97	20.51	16.60
TSRw (1~2mm)	80.61	85.10	18.14	14.37
TSRw (2~3mm)	81.28	85.66	17.47	13.82

Table S3. Elemental content of FCR at different corrosion depths after 28 days of gas phase and water phase corrosion.

Elements	C		O		S		Ba	
**Proportion /%**	**Weight**	**Atom**	**Weight**	**Atom**	**Weight**	**Atom**	**Weight**	**Atom**
FCR_0	41.59	64.39	19.07	22.16	3.32	1.93	17.75	2.40
FCRg (0~1mm)	49.40	69.89	20.40	21.67	3.14	1.66	16.76	2.07
FCRg (1~2mm)	50.49	72.55	16.88	18.20	3.70	1.99	18.58	2.34
FCRg (2~3mm)	52.07	73.05	18.11	19.07	3.67	1.93	17.86	2.19
FCRw (0~1mm)	52.12	69.43	22.64	22.64	2.82	1.41	10.87	1.27
FCRw (1~2mm)	51.47	70.70	21.00	21.66	3.38	1.74	15.09	1.81
FCRw (2~3mm)	49.39	68.93	21.59	22.62	3.28	1.72	14.81	1.81

In contrast, the elemental composition of RC and PC samples changed significantly after corrosion. In particular, the content of major elements such

as C, O, Si, and Ca showed obvious differences under different corrosion depths. This change is mainly due to the dual effects of carbonization and neutralization. In the process of carbonization and neutralization, calcium hydroxide is gradually converted into calcium carbonate by reacting with carbon dioxide, resulting in changes in the proportion of related elements. The leaching decalcification changes calcium carbonate into soluble calcium bicarbonate, which further aggravates the loss of calcium carbonate components and causes the element content to fluctuate more violently.

Table S4. Elemental content of RC at different corrosion depths after 28 days of gas phase and water phase corrosion.

**Elements**	**C**		**O**		**Si**		**Ca**	
**Proportion /%**	**Weight**	**Atom**	**Weight**	**Atom**	**Weight**	**Atom**	**Weight**	**Atom**
RC_0	31.26	43.76	41.97	44.10	5.14	3.08	21.63	9.07
RCg (0~1mm)	35.30	50.50	32.24	34.63	5.26	3.22	27.19	11.66
RCg (1~2mm)	50.18	65.30	24.78	24.21	4.42	2.46	20.62	8.04
RCg (2~3mm)	49.91	64.75	25.81	25.14	4.04	2.24	20.24	7.87
RCw (0~1mm)	37.62	51.75	33.40	34.49	10.25	6.03	18.73	7.72
RCw (1~2mm)	39.38	54.74	30.51	31.84	4.91	2.92	25.20	10.50
RCw (2~3mm)	31.74	45.08	37.74	40.24	9.28	5.63	21.24	9.04

Table S5. Elemental content of PC at different corrosion depths after 28 days of gas phase

and water phase corrosion.

Elements	C		O		Si		Ca	
Proportion /%	Weight	Atom	Weight	Atom	Weight	Atom	Weight	Atom
PC_0	9.43	15.44	52.38	64.39	6.81	4.77	31.38	15.40
PCg (0~1mm)	11.49	18.97	47.53	58.89	8.84	6.24	32.14	15.90
PCg (1~2mm)	12.73	21.23	43.97	55.03	9.87	7.04	33.43	16.70
PCg (2~3mm)	12.65	21.09	42.50	53.19	15.55	11.08	29.30	14.64
PCw (0~1mm)	7.58	14.63	35.88	52.02	2.60	2.14	53.94	31.21
PCw (1~2mm)	7.67	14.56	36.12	51.47	8.22	6.67	47.99	27.30
PCw (2~3mm)	8.17	15.16	39.68	55.30	2.22	1.76	49.93	27.78

References

1. GB/T 29172-2012; *Core analysis method*. National Oil and Gas Standardization Technical Committee: Beijing, China, 2012.
2. GB/T 2567-2021; *Test method for properties of resin castings*. National fiber-reinforced plastics standardization Technical Committee: Beijing, China, 2021.
